# The db/db Mouse: A Useful Model for the Study of Diabetic Retinal Neurodegeneration

**DOI:** 10.1371/journal.pone.0097302

**Published:** 2014-05-16

**Authors:** Patricia Bogdanov, Lidia Corraliza, Josep A. Villena, Andrea R. Carvalho, José Garcia-Arumí, David Ramos, Jesús Ruberte, Rafael Simó, Cristina Hernández

**Affiliations:** 1 Diabetes and Metabolism Research Unit, Vall d’Hebron Research Institute, Universitat Autònoma de Barcelona, Barcelona, Spain; 2 Centro de Investigación Biomédica en Red de Diabetes y Enfermedades Metabólicas Asociadas (CIBERDEM), Instituto de Salud Carlos III (ISCIII), Madrid, Spain; 3 Laboratory of Metabolism and Obesity, Vall d’Hebron Research Institute, Universitat Autònoma de Barcelona, Spain; 4 Ophthalmologic Research Laboratory. Vall d’Hebron Research Institute. Universitat Autònoma de Barcelona, Barcelona, Spain; 5 Red Temática de Investigación en Oftalmolología (OFTARED), Instituto de Salud Carlos III (ISCIII), Madrid, Spain; 6 Department of Animal Health and Anatomy, Faculty of Veterinary Sciences, Universitat Autònoma de Barcelona, Barcelona, Spain; Queen's University Belfast, United Kingdom

## Abstract

**Background:**

To characterize the sequential events that are taking place in retinal neurodegeneration in a murine model of spontaneous type 2 diabetes (db/db mouse).

**Methods:**

C57BLKsJ-db/db mice were used as spontaneous type 2 diabetic animal model, and C57BLKsJ-db/+ mice served as the control group. To assess the chronological sequence of the abnormalities the analysis was performed at different ages (8, 16 and 24 weeks). The retinas were evaluated in terms of morphological and functional abnormalities [electroretinography (ERG)]. Histological markers of neurodegeneration (glial activation and apoptosis) were evaluated by immunohistochemistry. In addition glutamate levels and glutamate/aspartate transporter (GLAST) expression were assessed. Furthermore, to define gene expression changes associated with early diabetic retinopathy a transcriptome analyses was performed at 8 week. Furthermore, an additional interventional study to lower blood glucose levels was performed.

**Results:**

Glial activation was higher in diabetic than in non diabetic mice in all the stages (p<0.01). In addition, a progressive loss of ganglion cells and a significant reduction of neuroretinal thickness were also observed in diabetic mice. All these histological hallmarks of neurodegeneration were less pronounced at week 8 than at week 16 and 24. Significant ERG abnormalities were present in diabetic mice at weeks 16 and 24 but not at week 8. Moreover, we observed a progressive accumulation of glutamate in diabetic mice associated with an early downregulation of GLAST. Morphological and ERG abnormalities were abrogated by lowering blood glucose levels. Finally, a dysregulation of several genes related to neurotransmission and oxidative stress such as UCP2 were found at week 8.

**Conclusions:**

Our results suggest that db/db mouse reproduce the features of the neurodegenerative process that occurs in the human diabetic eye. Therefore, it seems an appropriate model for investigating the underlying mechanisms of diabetes-induced retinal neurodegeneration and for testing neuroprotective drugs.

## Introduction

Diabetic retinopathy (DR) is the most common complication of diabetes and one of the leading causes of preventable blindness [1]. Current treatments for DR are applicable only at advanced stages of the disease and are associated with significant adverse effects [2], [3]. Therefore, new pharmacological treatments for the early stages of the disease are needed. However, the mechanisms involved in the onset of DR are still poorly understood.

Emerging evidence suggests that retinal neurodegeneration is an early event in the pathogenesis of DR [4]–[8] which participates in the microcirculatory abnormalities that occur in DR [9]–[13]. Consequently, new therapeutic strategies based on neuroprotection have been proposed [14]–[15].

The experimental model currently used to study retinal neurodegeneration in DR is the rat with streptozotocin-induced diabetes (STZ-DM). However, since STZ is neurotoxic itself [16], a debate has arisen regarding the appropriateness of this model for examining retinal neurodegeneration shortly after STZ administration. A second rodent model, the Ins2Akita (Akita) mouse, which contains a dominant point mutation in the gene encoding for insulin-2 that induces spontaneous type 1 diabetes in the B6 mouse strain, reproduces some findings of the neurodegenerative process that occurs in the human diabetic retina [17]. However, both STZ-DM and Akita mouse are models of type 1 diabetes and further characterization of the neurodegenerative process in type 2 models is needed.

In recent years the C57BL/KsJ-*db/db* mouse has been used as a spontaneous diabetic model of type 2 diabetes to investigate the pathogenesis of DR [17]–[23]. The C57BL/KsJ-*db/db* mouse carries a mutation in the leptin receptor gene and is a well-established model of obesity-induced type 2 diabetes. Several authors have reported the presence of retinal neurodegeneration (apoptosis, glial activation and retinal thinning) in this model [19], [22]. Therefore, C57BL/KsJ-*db/db* seems appropriate for investigating the underlying mechanisms of retinal neurodegeneration associated with diabetes and for testing new drugs. However, the characterization of the retinal neurodegenerative process and its functional consequences in db/db mice is far from being completed. In addition, whether neurodegeneration can be attributed to genetic factors rather than to diabetes is a question which remains to be elucidated.

In the present study we have characterized the neurodegenerative process that occurs in the retina of C57BL/KsJ-*db/db* mice by examining morphological, biochemical and functional abnormalities in a sequential manner (8, 16, 24 weeks). Moreover, a transcriptomic analysis in 8-week old diabetic mice was performed to identify new potential causative candidates of DR. In addition, we have demonstrated that the neurodegenerative process is significantly arrested after blood glucose levels have been lowered. Overall, our results suggest that C57BL/KsJ-*db/db* reproduces the neurodegenerative features that occur in the human diabetic eye, and is an appropriate experimental model for studying the mechanisms involved in diabetes-induced retinal neurodegeneration.

## Methods

### Animals

A total of 90 C57BL/KsJ-*db/db* male mice obtained from Harlan Laboratories, Inc. were divided into two groups: 45 non-diabetic (db/+) and 45 diabetic mice (db/db). To assess the chronological sequence of the retinal abnormalities associated with diabetes, 15 diabetic mice (db/db) were compared with 15 age-matched non-diabetic mice (db/+) at different ages (8, 16 and 24 weeks). Blood glucose concentrations were measured from the tail vein (glucose assay kit; Abbott). Mice were housed under controlled conditions of temperature (20°C) and humidity (60%) with a 12-hour light/dark cycle and had free access to food and water.

#### Interventional study

Diabetic mice (db/db) 8 weeks old received diet *ad* libitum (n = 10) or restrictive diet (normal chow diet restricted to 60% of total daily calories; n = 10) for 15 days. Ten non-diabetic mice matched by age served as control group. At day 15 the animals were euthanized by cervical dislocation and the eyes enucleated.

This study was approved by the Animal Care and Use Committee of VHIR (Vall d’Hebron Research Institute). All the experiments were performed in accordance with the tenets of the European Community (86/609/CEE) and ARVO (Association for Research in Vision and Ophthalmology).

### Electroretinography

Before retinal electrophysiological tests, the animals were dark-adapted (12 hours overnight) and anesthetized under a dim red light with a 2% isoflurane/O2 mixture. Pupils were dilated with topic 1% tropicamide and ciclopegic was applied on the corneal surface. Full field electroretinography (ERG) recordings were measured using an HMsERG (Ocuscience) with two recording channels. Recordings were measured from corneal electrodes attached to the corneas by a lens embedded in 1% methylcellulose to avoid cornea dehydratation. Two needle probes were inserted for reference subcutaneously between each jaw and a grounding probe were inserted into the base of the tail. Then their noses were inserted into a mini Ganzfeld flash photo-stimulator with white LED.

Scotopic ERG stimuli were simultaneously recorded from both eyes of dark-adapted (12–16 hours) mice. Light stimuli were delivered via a Ganzfeld light source with flash intensities from 30 to 30000 mcd.s/m^−2^. Responses were amplified 5000X, high-pass filtered with a 10-Hz cutoff frequency, and low-pass filtered at 300 Hz using an amplifier. The ERG voltage and stimulus- monitor signals were digitalized with hardware (HMsERG) and software (HMsERG View) from Ocuscience. Data were recorded at either 0.2 or 0.5 ms/pt. A stimulus set consisted of 3 to 20 responses at the same wavelength and intensity of light. The oscillatory potentials (OPs) were isolated by a band-pass filtering the retinal response between 34 and 300 Hz. We chose 34 Hz as a cutoff frequency to avoid any loss of signal power, especially for the slower OPs of diabetic animals. OPs were isolated for a light stimulus of 3000 mcd.s/m^−2^.

The amplitude and implicit time of the ERG a-and b-waves were measured at the maximum negative and positive peaks of the recordings with respect to the baseline before stimulation. As recommended by the ISCEV (International Society for Clinical Electrophysiology of Vision) [24] OP amplitudes were measured from the negative peak to the next positive peak whereas OP latencies were measured at the positive peaks. We added up OP amplitudes (ΣOP amplitude) and implicit time (ΣOP implicit time) for the first 5 OPs.

### Tissue Processing

Mice were euthanized by cervical dislocation. The eyes were immediately enucleated and the neuroretina was separated. The neuroretina from one of the eyes was frozen in liquid nitrogen and stored at –80°C for protein assessments. The other eye was flash frozen in Tissue Freezing Medium (TFM, Electron Microscopy Sciences), by immersion in liquid nitrogen, and cryosectioned at 8 µm through the dorsal/ventral plane. Sections were mounted on slides and stored at −80°C. These sections were prepared for the assessment of retinal morphology, evaluation of GFAP and TUNEL immunoreactivity. For caspase-3 immunohistochemistry, the eyes were fixed overnight in 10% neutral buffered formalin and embedded in paraffin. Ocular globes embedded in paraffin were sectioned (3 µm) along the eye axis.

### Neurodegeneration Measurements

#### Retinal morphometry

Microscopic evaluation of retinas included scanning tissue sections to evaluate morphology followed by systematic morphometric analysis. The sections were stained with hematoxylin and eosin (H&E). Images of H&E sections were captured with a microscope (Olympus, Lake Success, NY) using the program Image J for quantification. The measurements were taken at two peripheral and three central regions of the retina and were examined to ensure similar locations of measurements for all eyes. Sections through the posterior eye segment were defined as central retina when the plane passed through the optic nerve or at less than 300 µm from the optic head rim. The remaining sections from both sites of the optic nerve were considered as peripheral retina. Image analysis of ten sections of each region were used to quantify total retinal thickness, the thickness of the inner nuclear layer (INL), outer nuclear layer (ONL) and cell number per mm^2^ in ganglion cell layer (GCL). These measurements were performed by two of the investigators (P.B and L.C).

#### Immunohistochemical analysis for glial activation assessment

Glial activation was evaluated by fluorescence microscopy using specific antibodies against GFAP (Glial fibrillar acidic protein). Sections were fixed in acid methanol (−20°C) for 2 min, followed by three washes with PBS, 5 min each. Sections were permeabilized with TBS-Triton X-100 0,025% and were incubated in blocker (1% BSA, and 10% goat serum in PBS) for 2 hours at room temperature. Sections were then incubated with rabbit anti- GFAP (Abcam Ltd, Cambridge, U.K.) (1∶500 dilution prepared in blocking solution) overnight at 4°C in a humid atmosphere. After three washes in PBS, 5 min each, the sections were incubated with secondary antibody Alexa 488 goat-anti-rabbit (Life Technologies S.A, Madrid, Spain) (1∶200 dilution prepared in blocking solution). The sections were washed three times in PBS, counterstained with Hoesch and mounted with Mounting Medium Fluorescence (Prolong, Invitrogen) and mounted with a coverslip. Comparative digital images from diabetic and control samples were recorded with an Olympus microscope using identical brightness and contrast settings.

To evaluate the degree of glial activation we used a scoring system based on extent of GFAP staining previously used [25]. The scoring system was as follows: Müller cell endfeet region/GCL only (score 1); Müller cell endfeet region/GCL plus a few proximal processes (score 2); Müller cell endfeet plus many processes, but not extending to ONL (score 3); Müller cell endfeet plus processes throughout with some in the ONL (score 4); Müller cell endfeet plus lots of dark processes from GCL to outer margin of ONL (score 5).

#### Immunohistochemical analysis for apoptosis assessment

Apoptosis was evaluated using the TUNEL (Terminal Transferase dUTP Nick-End Labeling) method coupled with fluorescein (DeadEnd Fluorometric TUNEL System kit; PROMEGA, USA) with Hoechst 33342, Trihydrochloride, Trihydrate (Molecular Probes) staining. Cryosections of retina were permeabilised by incubation for 2 min on ice with 0.1% Triton X-100 in 0.1% sodium citrate, freshly prepared. Apoptotic cells were identified using green fluorescence [Alexa Fluor 594 goat-anti-rabbit (Invitrogen) (1∶200 dilution prepared in blocking solution with 5% BSA)]. For evaluation by fluorescence microscopy an excitation wavelength in the range of 450–500 nm (e.g., 488 nm) and detection in the range of 515–565 nm (green) was used.

#### Immunohistochemistry for caspase-3

Paraffined sections were rehydrated and washed in 0.01-M phosphate buffered saline (PBS). Then, they were incubated over night at 4°C with a rabbit anti-cleaved caspase-3 antibody (Cell Signalling Technology, Inc., Danvers, USA) at 1∶300 dilution. Then, ocular sections were washed in PBS and incubated with the specific secondary antibody biotinylated anti-rabbit IgG (1∶100) (Vector Laboratories, Burlingame, USA). Once washed in PBS, a streptavidin Alexa Fluor 488 conjugate (Molecular Probes-Life Technologies, Grand Island, USA) at 1∶100 dilution was used to detect cleaved caspase-3 immunolabelling; the incubation was made over night at 4°C. Nuclear counterstaining with Hoescht stain solution (Sigma-Aldrich Chemie, Buchs, Switzerland) was performed for microscopic analysis with the laser scanning confocal microscope (TCS SP2; Leica Microsystems, Wetzlar, Germany). Negative control was carried out by omitting the primary antibody. Specific labeling of cleaved caspase-3 antibody in mouse jejunal paraffin sections was used as positive control.

#### Transmission electron microscopy analysis

One mm^3^ retinal fragments were dissected from four 8 week-old db/db mice. Retinas from four age-matched db/+ mice were used as control retinas. Retinal fragments were fixed in 2.5% glutaraldehyde and 2% paraformaldehyde, post fixed in 1% osmium tetroxide, stained in aqueous uranyl acetate, dehydrated and embedded in spurr resin. Ultrathin sections (70 nm) were stained with lead citrate and examined under transmission electron microscopy (Jeol 1400; Jeol Ltd., Tokyo, Japan).

### Glutamate Quantification

Quantification of glutamate was performed by liquid-chromatography coupled to mass spectrometry (LC-MS/MS). Chromatographic separation was performed on an Agilent 1200 series (Waldbronn, Germany) using an Ascentis Express HILIC column, 50×2.1 mm with 2.7 Rm particle size from Supelco (Belfonte, PA) maintained at 25°C throughout the analysis, a mobile phase acetonitrile and water (50 mM ammonium acetate) with a flow rate of 0.6 mL min-1. The volume injected was 10 RL. The mobile phase involved a gradient starting at 87% of ACN which was maintained for 3 minutes. Then, from min 3 to 10 the ACN content was decreased to 20% and increased again to 87% at min 12.5. Glutamate was eluted at 6.5 minutes. The mass detection system was an Agilent 6410 Triple Quad (Santa Clara, CA) using positive electrospray ionization with a gas temperature of 350°C, gas flow rate of 12 L min-1, nebulizer pressure of 45 psi, capillary voltage of 3500 V, fragmentor of 135 V and collision energy of 10 V.

### Immunohistochemistry for GLAST

Glutamate/aspartate transporter (GLAST) and L-glutamate was evaluated by fluorescence microscopy using specific antibodies. Sections were incubated in blocking solution (3% BSA, Tween 0,05% PBS) for 1 h. at room temperature followed by incubation with primary antibody rabbit anti-GLAST (EAAT1) (1∶100, Abcam ab416, Cambridge, UK). After washing, sections were incubated with a fluorescent anti-rabbit ALEXA 594 as a secondary antibody (Life Technologies S.A, Madrid, Spain) in blocking solution for 1 h, washed, nuclei were stained with Hoechst and mounted in Mounting Medium Fluorescence (Prolong, Invitrogen) with a coverslip. Fluorescence intensity of images was quantified by ImageJ.

### DNA Microarrays

Gene expression profiling analysis in retinas of 8-week old diabetic (db/db) mice and non-diabetic (db/+) controls was performed using Mouse Gene 1.0 ST DNA arrays (Affymetrix, UK). For this purpose, total RNA was first isolated from retinas (n = 4/group) using the RNeasy Mini Kit (Qiagen, Germany) and 200 ng were then used to synthesize sense ssDNA with the Ambion WT Expression Kit (Life Technologies, UK). Next, ssDNA was fragmented, labeled and hybridized onto DNA microarrays using the GeneChip WT Terminal Labeling and Hybridization Kit and the GeneTitan platform (Affymetrix, UK), following the manufacturer’s instructions. Microarray Analysis Suite 5.0 software was used to process the microarray images and analysis of the data obtained was performed by the Statistics and Bioinformatics Unit of the Vall d’Hebron-Research Institute using the open source software Bioconductor.

### Gene Expression

Real-time quantitative PCR was used to quantify relative transcript levels in retinas of 8-week old diabetic and control mice. 400 ng of total RNA were used to synthesize cDNA by using SuperScript II reverse transcriptase (Life Technologies, USA) and oligo(dT) primers. Quantitative PCR was performed by using gene-specific primers and SYBR Green in an ABI PRISM 7500 Sequence Detection System (Applied Biosystems, UK). Relative mRNA expression was calculated according to the 2^−ΔΔCT^ threshold method, using cyclophilin A as a reference gene [26].

### Statistical Analysis

Normal distribution of the variables was evaluated using the Kolmogorov-Smirnov test. Comparisons of continuous variables were performed using the paired and unpaired Student t-test. Levels of statistical significance were set at p<0.05.

## Results

### Blood Glucose Levels

**Figure 1:**
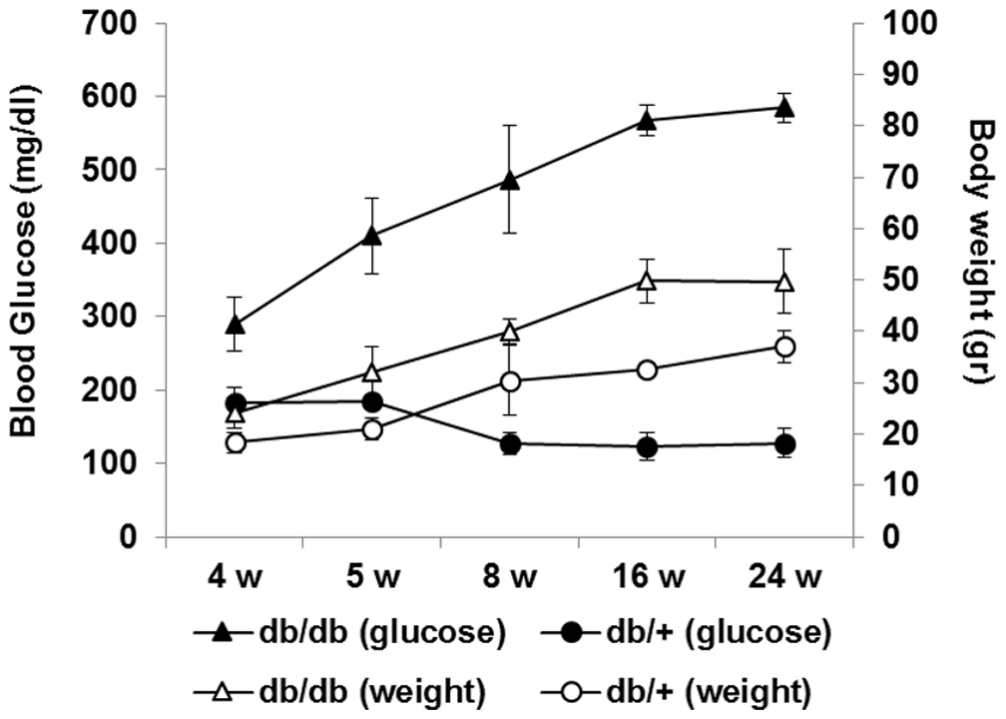
Blood glucose levels (black marks) and body weight (white marks) in db/+ (circles; n = 12) and db/db mice (triangles; n = 12). Values are expressed as mean ± SD. *p<0.05; **p<0.001.

The diagnosis of diabetes was based on blood glucose levels. The mice with blood glucose level greater than 250 mg/dl were confirmed as diabetic mice. In the control group (db/+) blood glucose levels were <150 mg/dl during the follow-up whilst all db/db mice presented blood glucose >250 mg/dl at 4 weeks of age. Animal body weight and glucose levels are shown in Figure 1. We observed that hyperglycemia in diabetic mice runs in parallel with a significant increase of weight.

### ERG Abnormalities

**Figure 2:**
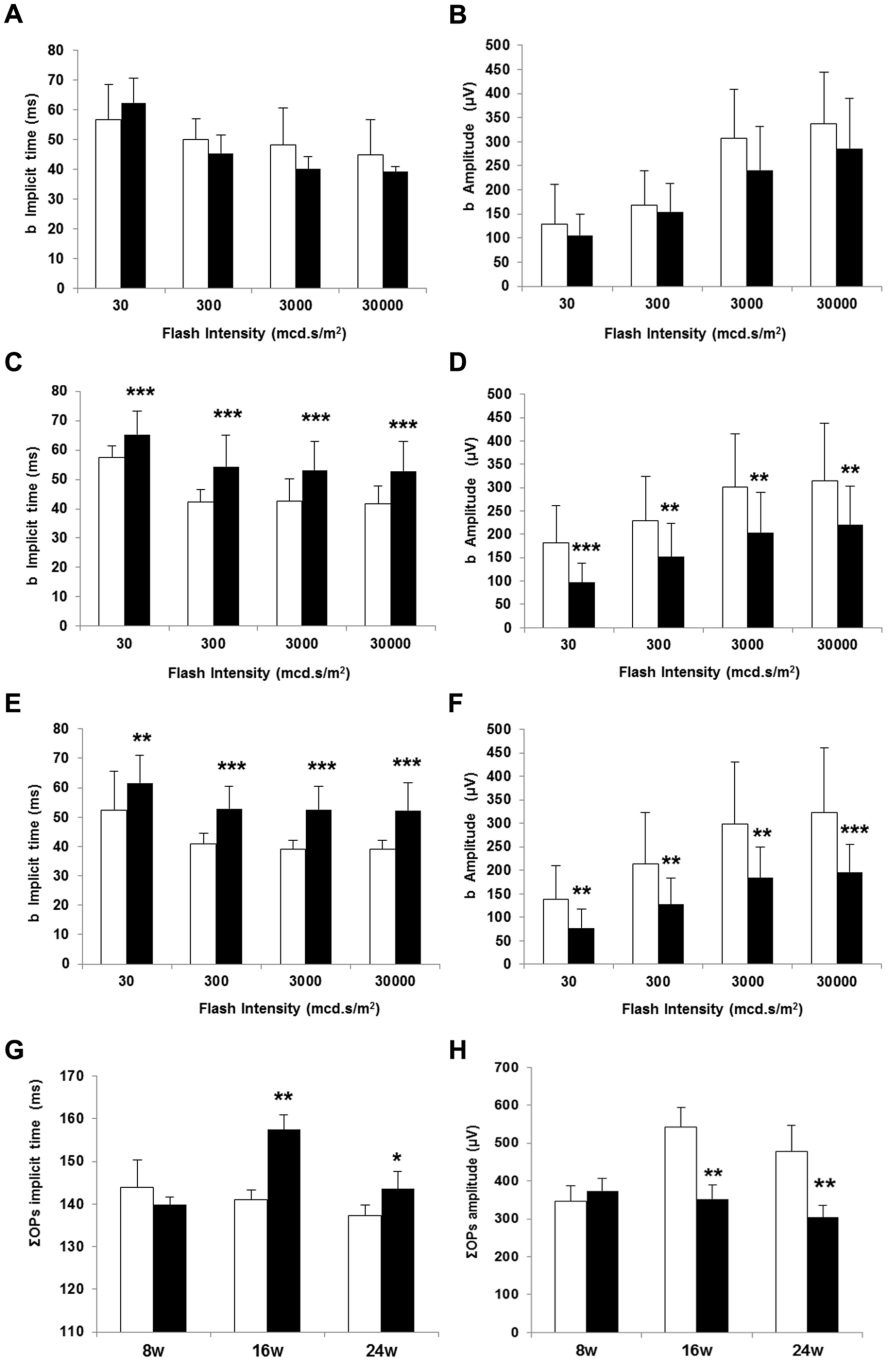
b-wave implicite time (ms) in non diabetic and diabetic mice at 8 (A), 16 (C) and 24 weeks (E). ΣOPs implicit time (ms) (G). b-wave amplitude (µV) in non diabetic and diabetic mice at 8 (B), 16 (D) and 24 weeks (F). ΣOPs amplitude (µV) (H). White bars: non diabetic mice. Black bars: diabetic mice. Data are expressed as mean ± SD. *p<0.05; **p<0.01;***p<0.001.

**Figure 3:**
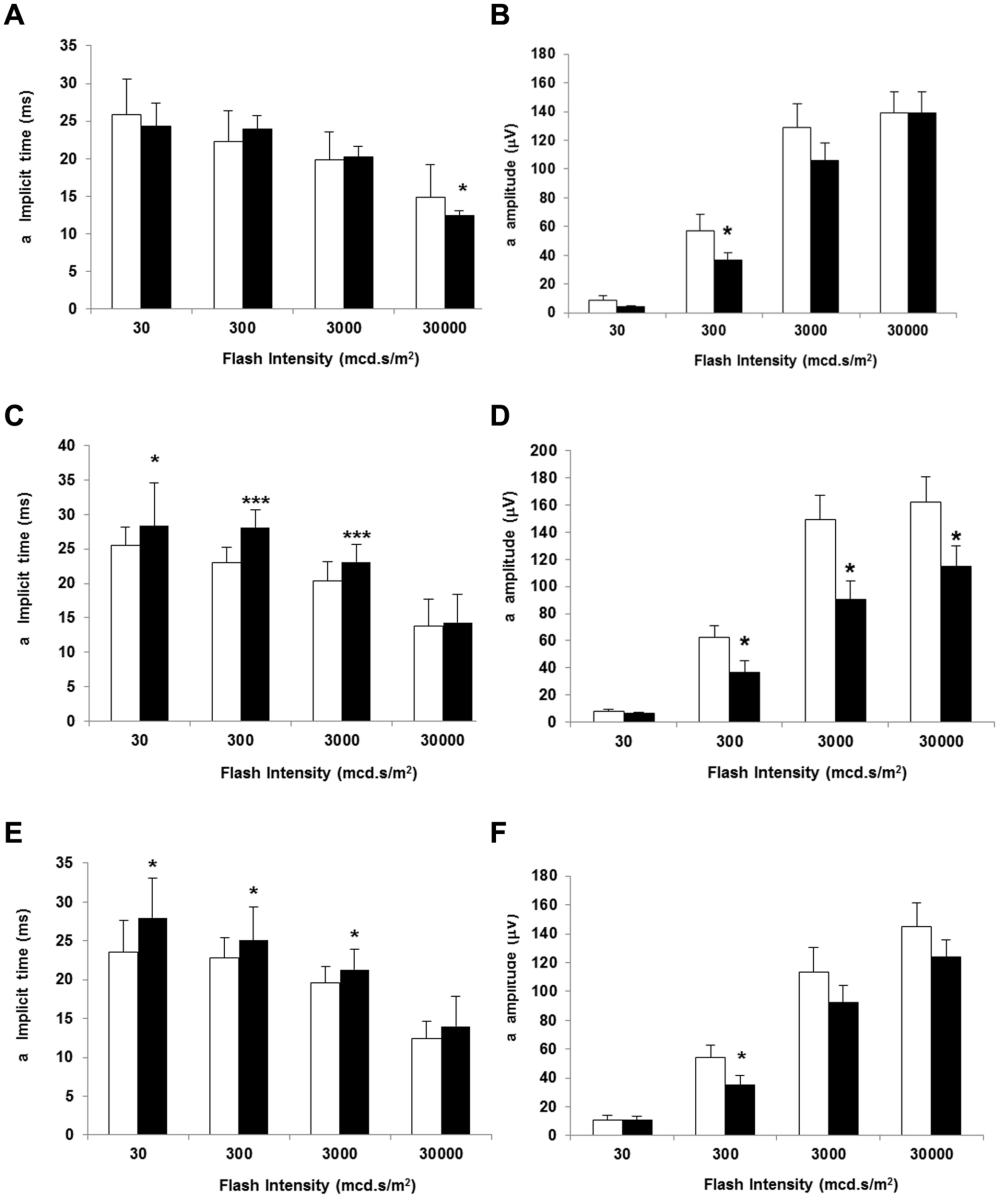
a-wave implicit time (ms) in non diabetic and diabetic mice at 8 (A), 16 (C) and 24 weeks (E). a-wave amplitude (µV) in non diabetic and diabetic mice at 8 (B), 16 (D) and 24 weeks (F). White bars: non diabetic mice. Black bars: diabetic mice. Data are expressed as mean ± SD. *p<0.05.

The average b-wave implicit times and b-wave amplitudes as a function of flash intensity are presented in Figure 2 (A–F). The b-wave implicit time significantly increased in diabetic mice at all flash intensities tested when compared with non-diabetic mice at 16 and 24 weeks. However, the implicit time was not significantly delayed in diabetic mice at 8 weeks of age. In addition, b-wave amplitude was significantly reduced in diabetic mice in comparison with non diabetic mice at 16 and 24 weeks but not at 8 weeks. The changes observed in amplitude and implicit time of the a-wave in diabetic mice in comparison with non-diabetic mice were less marked than that observed in b-wave (Figure 3).

The OPs amplitudes measured under scotopic conditions and the corresponding implicit times are shown in Figure 2 (G, H). We detected statistically significant differences (increase of implicit time and decrease of amplitude) at 16 and 24 weeks.

### Retinal Morphometry

**Figure 4:**
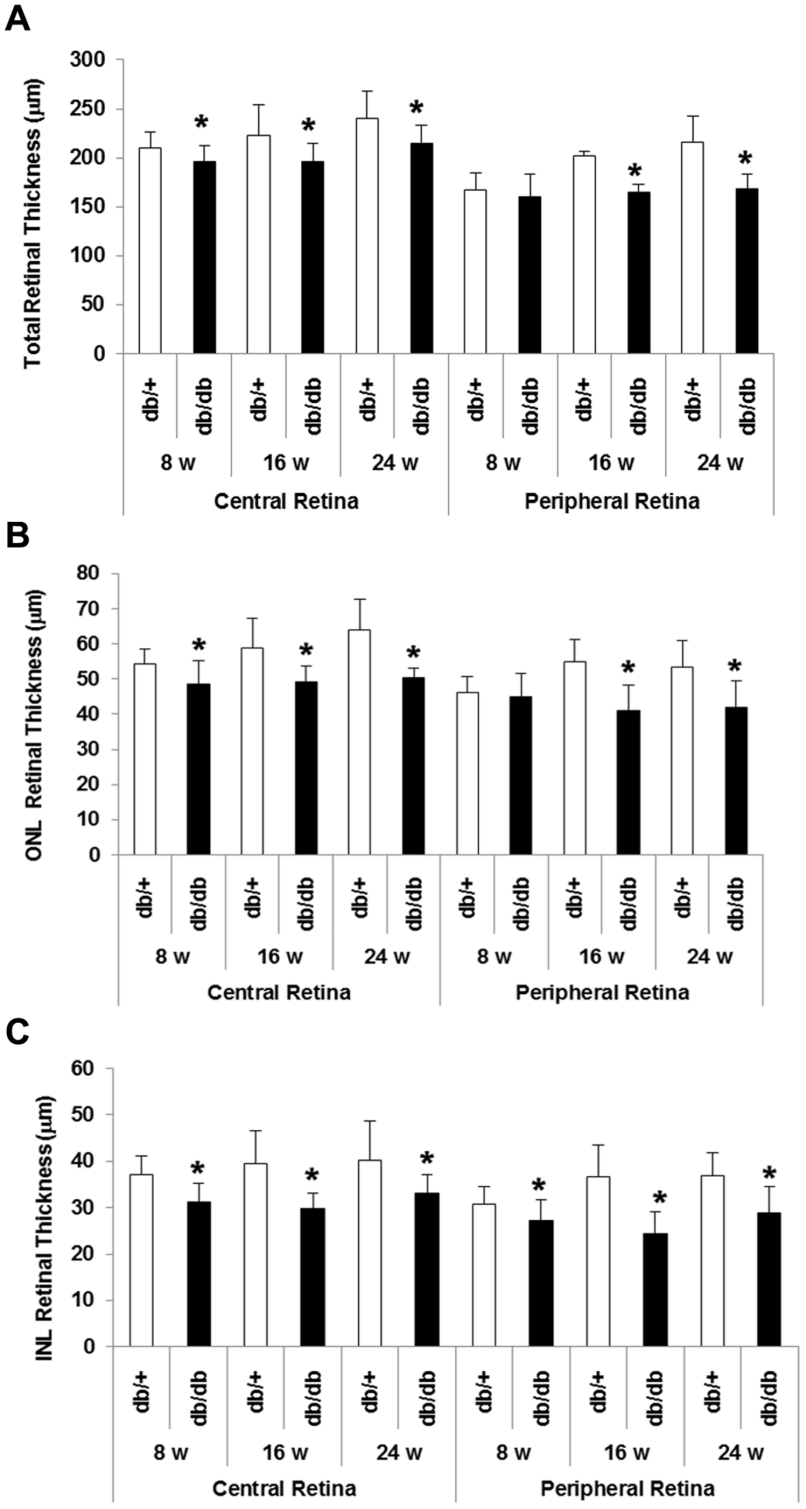
Thickness of total retina (A), outer nuclear layer (B) and inner nuclear layer (C). The measurements have been performed in the central retina and in peripheral retina. Results are expressed as mean ± SD. *p<0.05 between non diabetic (white bars) and diabetic (black bars) mice.

**Figure 5:**
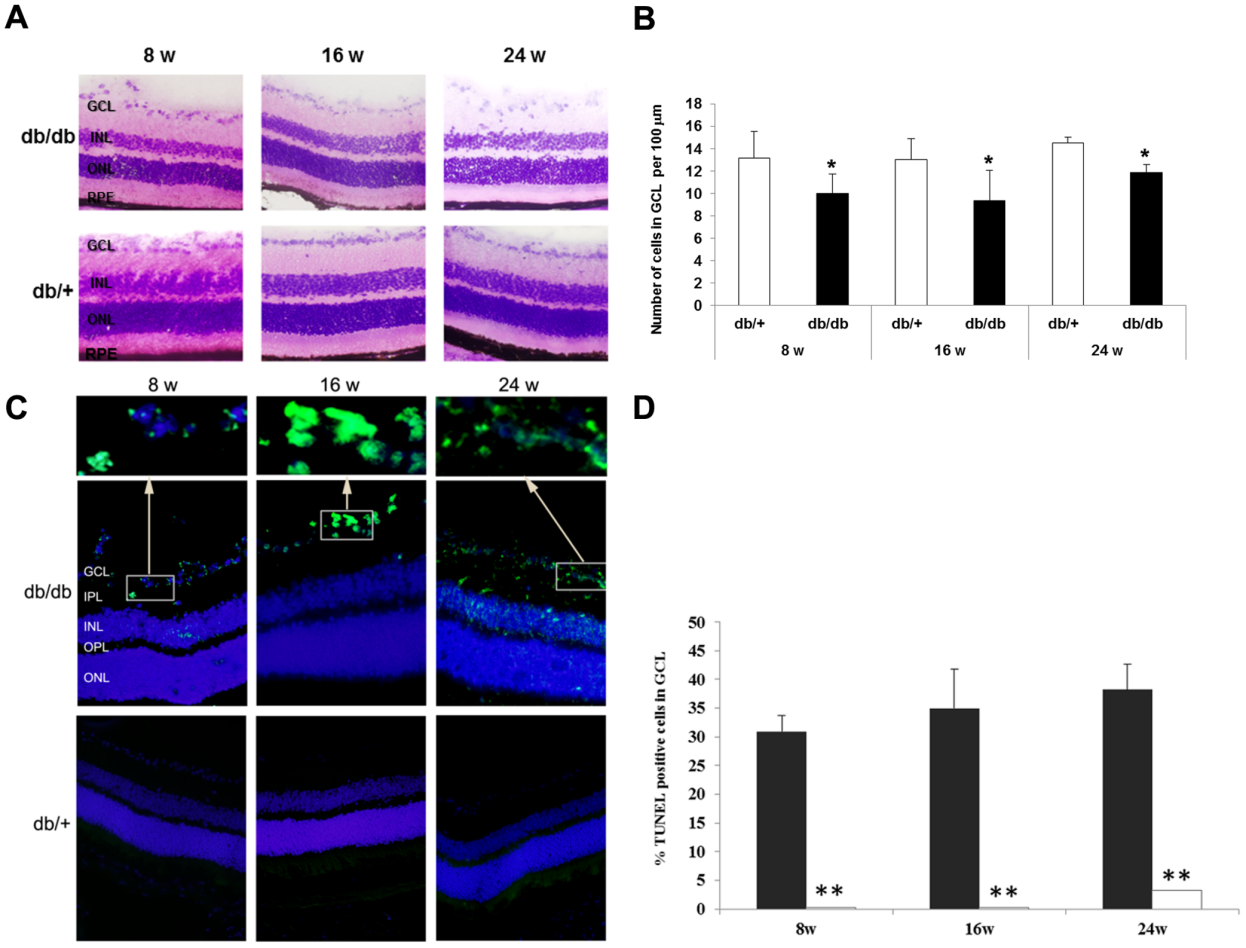
Images demonstrating the progressive loss of cells in retinal ganglion layer (GCL). A) Hematoxylin and eosin stained central retina in a representative case of a diabetic mouse (upper panel) and a non-diabetic mouse (lower panel) of 8, 16 and 24 weeks old. In the diabetic retina a loss of cells in GCL was observed. B) Cell number in GCL in control and diabetic mice in central retina. C) Comparison of TUNEL immunofluorescence (green) between representative samples from a diabetic (upper panel) and a non-diabetic mouse (lower panel) at 8 weeks, 16 and 24 weeks. Nuclei were labeled with Hoechst (blue). D) Percentage of TUNEL positive cells in the GCL in non-diabetic and diabetic mice at 8, 16 and 24 weeks (upper panel). ONL: outer nuclear layer; OPL: outer plexiform layer; INL: inner nuclear layer; IPL: inner plexiform layer; GCL: ganglion cell layer. Results are expressed as mean ± SD. White bars: non-diabetic mice; Black bars: diabetic mice. *p<0.05, **p<0.001 between non-diabetic and diabetic mice.

Measurements of retinal thickness in diabetic and in non diabetic mice at 8, 16 and 24 weeks are shown in Figure 2. Since postnatal growth of retinal thickness exists in C57BL mice until month 6 [27], [28], a progressive increase in the thickness of retinas from non-diabetic mice was observed whereas this was not the case in diabetic mice. Total retinal thickness (measured from inner limiting membrane to Bruch’s membrane) in both central and peripheral retina was significantly decreased in diabetic mice in comparison with non-diabetic mice at 16 and 24 weeks (Figure 4). Furthermore, a thickening in both ONL and INL (Figure 4) as well as a reduction in the number of cells in the GCL (Figure 5B) was observed in diabetic mice in comparison with non-diabetic mice at 8, 16 and 24 weeks.

### Neurodegeneration Features

**Figure 6:**
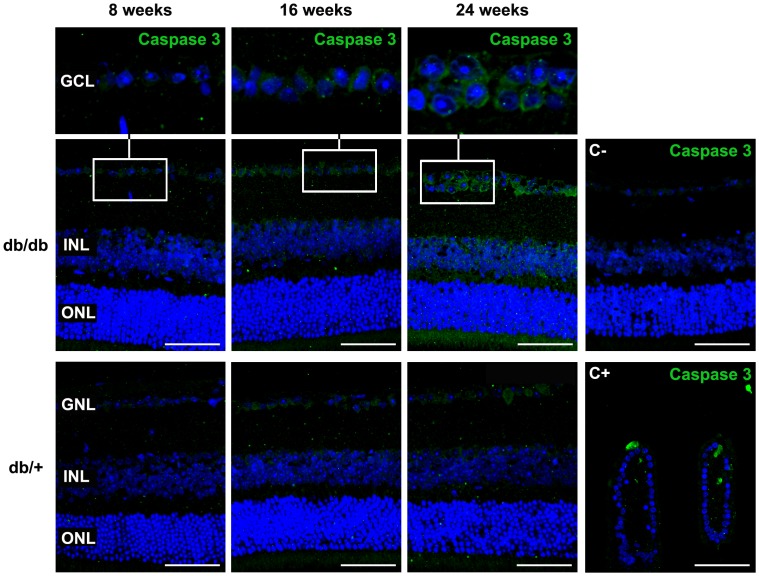
Comparison of cleaved caspase-3 immunofluorescence (green) between representative samples from a diabetic (upper panel) and a non-diabetic mouse (lower panel) at 8, 16 and 24 weeks. Insets show the increased expression of cleaved caspase-3 in cells residing in the ganglion cell layer (GCL) in db/db mice. Nuclei counterstained with Hoescht (blue). ONL: outer nuclear layer; INL: inner nuclear layer; C-: negative control; C+: positive control mouse jejunal villi. Scale bars: 40 µm.

A significant increase in TUNEL-positive inmunofluorescence was observed in diabetic mice in comparison with retinas from non diabetic mice at 8, 16 weeks and 24 weeks (Figure 5C). Since the TUNEL-positive cells were mainly localized in the GCL, we also counted the percentage of apoptotic cells in this layer, and a significant increase was found in diabetic mice in comparison with non-diabetic mice at 8, 16 and 24 weeks (Figure 5D). In addition, activated caspase-3 was found significantly higher in the retina of db/db mice in comparison with non-diabetic mice at 8, 16 and 24 weeks (Figure 6).

**Figure 7:**
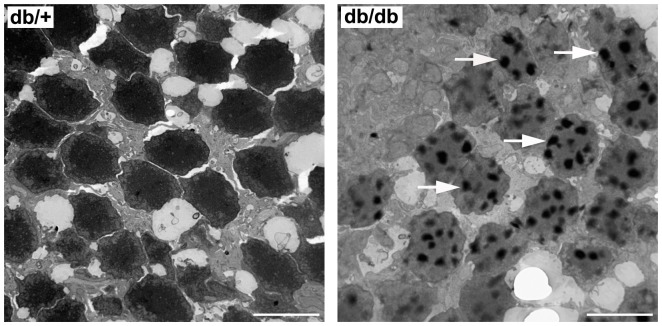
Transmission electron micrographs of photoreceptors in a representative case of a non-diabetic (left panel) and a diabetic mouse (right panel) 8 weeks old. Arrows indicate nuclear fragmentation. Scale bars: 6.71 µm.

Since in the ERG measurements we found a-wave abnormalities, which mainly indicate photoreceptor impairment, we wanted to examine whether apoptosis was also present in photoreceptors. For this purpose transmision electron microsocopy was used and striking DNA fragmentation was found in photoreceptors from db/db mice in comparison with non-diabetic mice (Figure 7).

**Figure 8:**
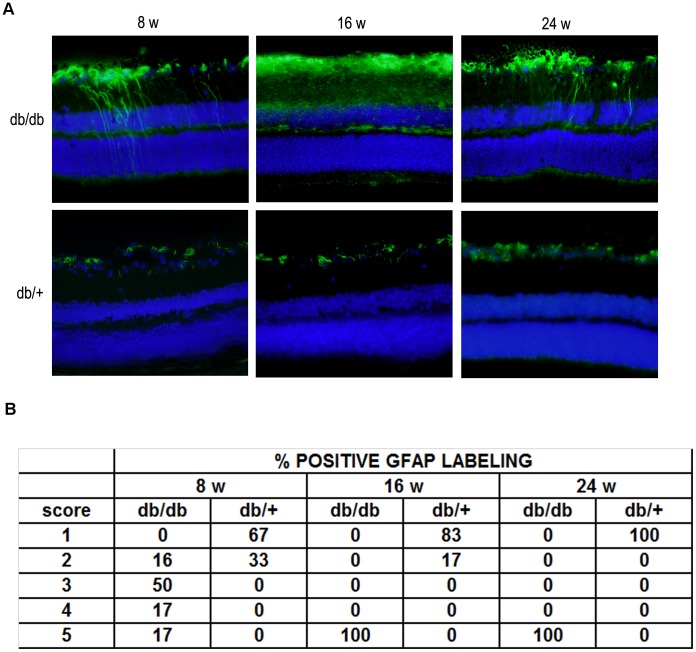
Glial activation. A) Comparison of GFAP immunofluorescence (green) between representative samples from a diabetic (upper panel) and a non-diabetic mouse (lower panel) at 8 weeks, 16 and 24 weeks. In the diabetic retina, the Müller cells’ endfeet show abundant GFAP immunofluorescence and the radial processes stain intensely throughout both the inner and outer retina. Nuclei were labeled with DAPI (blue). ONL: outer nuclear layer; INL: inner nuclear layer; GCL: ganglion cell layer. B) Quantification of glial activation based on extent of GFAP staining.

As expected, in non-diabetic mice GFAP expression was confined to the retinal GCL (GFAP score ≤2) (Figure 8). In contrast, in diabetic mice we observed the “reactive” diabetic phenotype characterized by upregulation of GFAP in Müller cells (GFAP score ≥2 at week 8 and score = 5 at 16 and 24 weeks).

All these features of neurodegeneration were more intense at 16 than at 8 weeks, but no significant differences between 16 and 24 weeks were observed.

### Effect of Lowering Blood Glucose on Retinal Neurodegeneration

**Figure 9:**
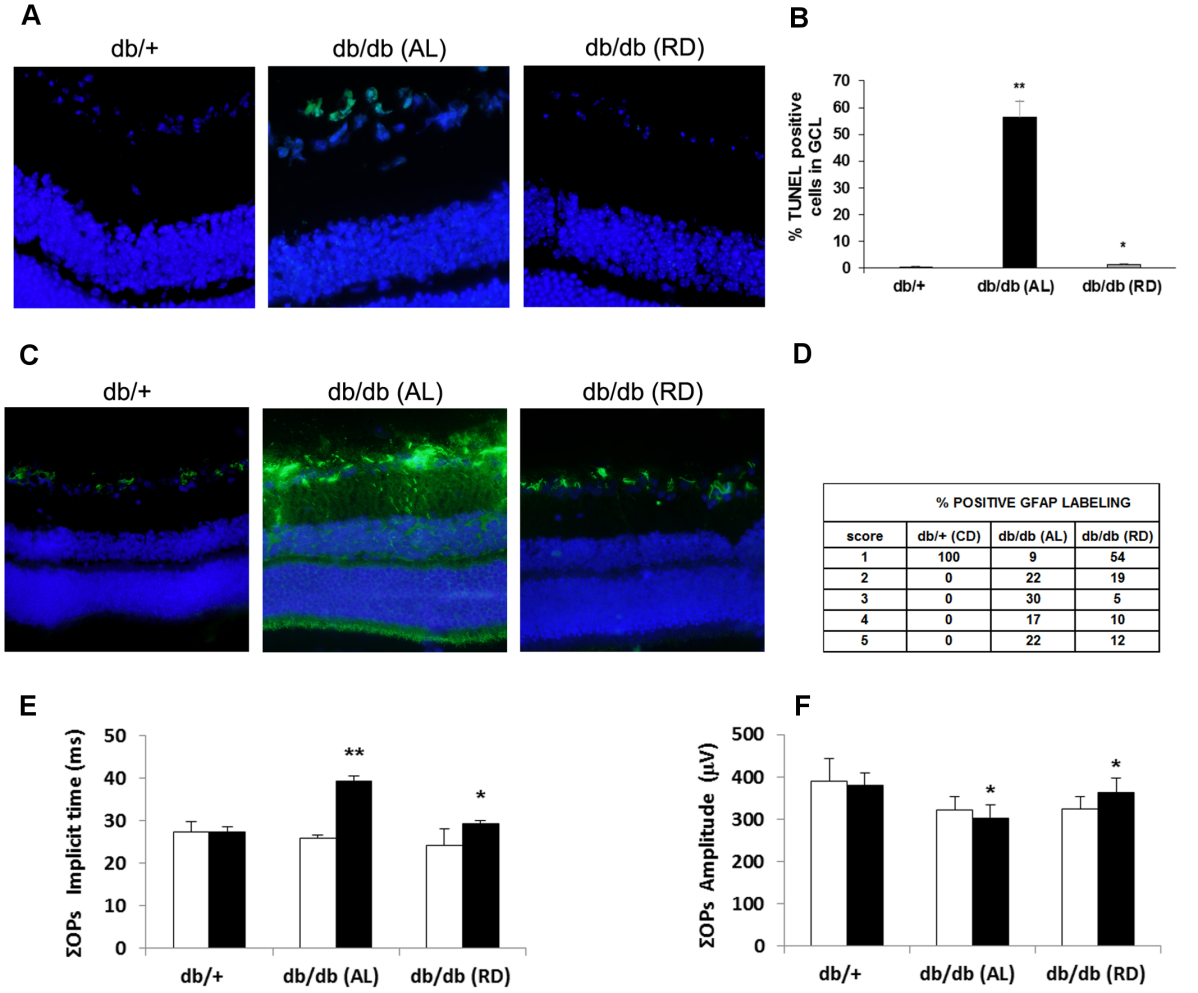
Beneficial effects of lowering blood glucose by using restrictive diet on retinal neurodegeneration in db/db mice. A) Comparison of TUNEL immunofluorescence (green) between representative samples from a diabetic mouse (db/db) *ad libitum* (AL) feeding (upper panel), a db/db mouse under dietary restriction (RD) (middle panel) and a control (db/+) mouse (lower panel) at 10 weeks. B) Percentage of TUNEL positive cells in the GCL in db/db mice ad libitum feeding (black bars), db/db mice under dietary restriction (gray bars) and control (db/+) mice (white bars) at 10 weeks. n = 10 each group. *p<0.05 in comparison with db/db feeding AL. **p<0.01 in comparison with db/+. C) Comparison of GFAP immunoreactivity (green) in the central retina between representative samples from a diabetic mouse (db/db) AL feeding (upper panel), a db/db mouse under dietary restriction (middle panel) and a control (db/+) mouse (lower panel) at 10 weeks. Nuclei were labeled with Hoechst (blue). ONL: outer nuclear layer; INL: inner nuclear layer; GCL: ganglion cell layer. D) Quantification of glial activation based on scoring system (see text) (n = 10 each group). E) ΣOPs implicit time (ms) and F) ΣOPs amplitude (µV). White bars: before diet intervention. Black bars: after diet intervention. Data are expressed as mean ± SD. *p<0.05; **p<0.01.

As expected, db/db mice fed for 15 days with restrictive diet presented lower weight and blood glucose levels than those mice fed with *ad libitum* diet (weigth: 32.5±2.8 gr vs. 42.2±2.5 gr; p<0.05; blood glucose: 318±27 mg/dL vs. 536±29 mg/dL, p<0.01). At this point (after 15 days of dietary restriction) a significantly lower GFAP immunofluorescence and a lower ratio of apoptosis in GCL were detected (Figures 9 A–D). Finally, ERG abnormalities were significantly arrested (Figure 9 E–F).

### Markers of Glutamate Pathway

**Figure 10:**
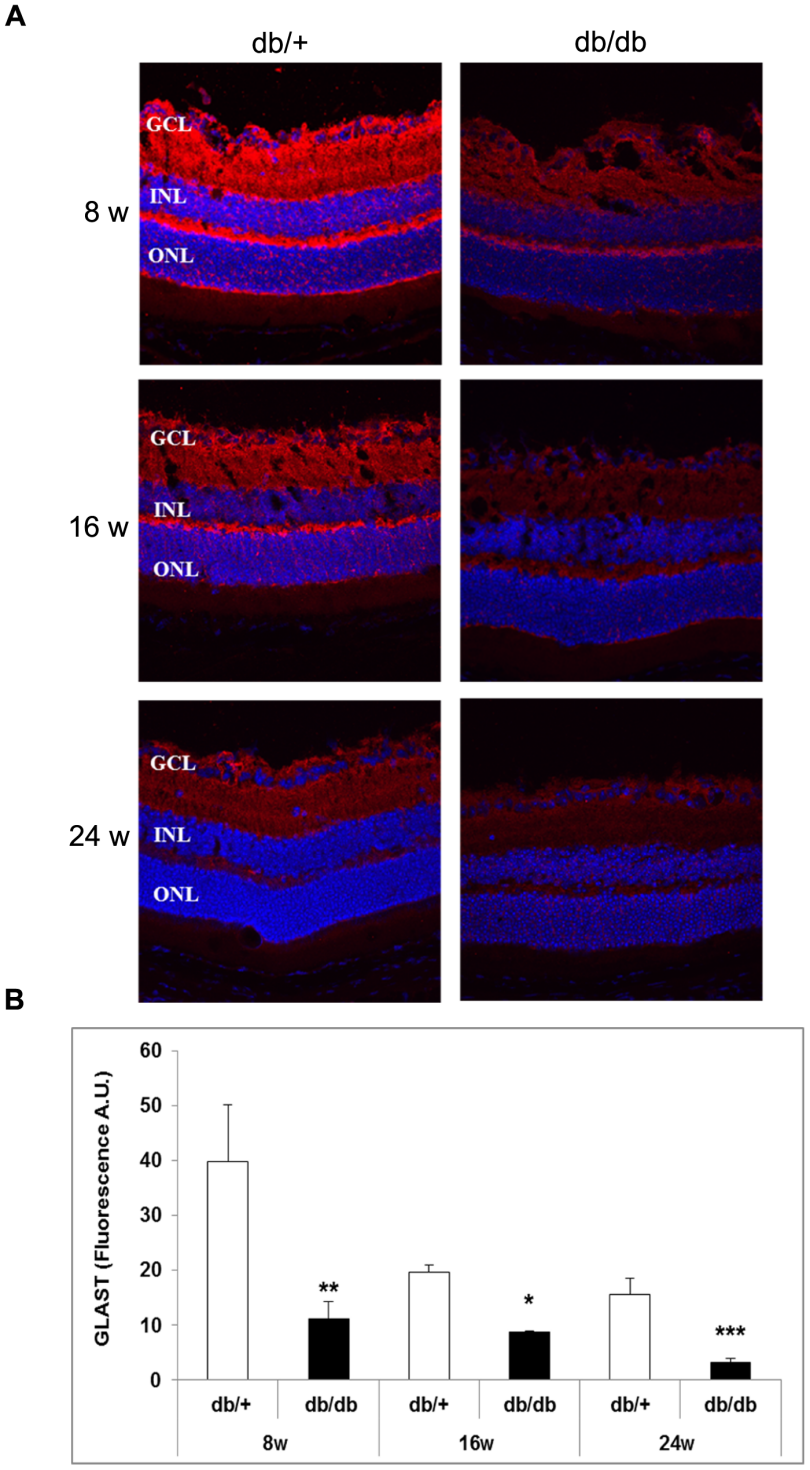
Comparison of GLAST immunofluorescence between representative samples from non-diabetic and diabetic mice. A) GLAST immunofluorescence (red) of representative samples from non-diabetic and diabetic mice at 8 weeks, 16 and 24 weeks. Nuclei were labeled with Hoechts (blue). ONL: outer nuclear layer; INL: inner nuclear layer; GCL: ganglion cell layer. B) Quantification of GLAST immunofluorescence in non-diabetic (white bars) and diabetic mice (black bars). A.U.: arbitray units. *p<0.05; **p<0.001 between non-diabetic and diabetic mice.

Glutamate levels (µM/g total protein) were higher in diabetic mice in comparison with non diabetic mice at 8 weeks (60±8 *vs.* 38±6; p<0.05), 16 weeks (108±12 vs. 56±10; p<0.05) and 24 weeks (125±11 vs. 57±9; p<0.05). The progressive increase of glutamate levels in diabetic mice run in parallel with a decrease in GLAST content. GLAST was significantly decreased in diabetic mice in comparison with the non diabetic mice (Figure 10).

### Transcriptomic Analysis

To unravel the molecular mechanisms involved in early retinal neurodegeneration besides glutamate and its metabolic pathways, we performed a genome-wide expression profiling analysis on total RNA isolated from retinas of 8-week old diabetic (db/db) and control littermate mice. After filtering for redundant and non-annotated gens, we found that 657 genes were differentially expressed in retina of diabetic mice (P<0.01). Of these genes, we found 316 genes to be up-regulated and 341 down-regulated. The main genes up-regulated and down-regulated are listed in Table 1 and Table 2, respectively. To gain insight into the biological function of the genes differentially expressed in retinas of diabetic mice, a gene enrichment analysis was performed. Interestingly, we found that Gene Ontology (GO) terms significantly enriched among the down-regulated genes fitted into categories related to synaptic transmission, with a particular abundance of terms related to glutamate transport and metabolism (Table 3). On the other hand, the GO terms significantly over-presented among the up-regulated genes corresponded to categories related to mitochondrial respiration and oxidative stress (Table 3).

**Table 1:** Genes up-regulated in retinas of diabetic mice.

ID	Symbol	Gene name	LogFC	P Value
71760	Agxt2l1	Alanine-glyoxylate aminotransferase 2-like 1	0.862	2.9E-07
19878	Rock2	Rho-associated, coiled-coil containing protein kinase 2	0.819	1.2E-06
66594	Uqcr11	Ubiquinol-cytochrome c reductase, complex III subunit XI	0.815	4.9E-06
11811	Apobec2	Apolipoprotein B mRNA editing enzyme, catalytic polypeptide-like 2	0.791	8.0E-08
20341	Selenbp1	Selenium binding protein 1	0.769	1.8E-04
57441	Gmnn	Geminin, DNA replication inhibitor	0.706	2.5E-05
15122	Hba-a1	Haemoglobin alpha, adult chain 1/2	0.691	1.4E-03
16668	Krt18	Keratin 18	0.691	9.3E-09
11815	Apod	Apolipoprotein D	0.677	5.4E-03
110880	Scn4a	Sodium channel, voltage-gated, type IV, alpha subunit	0.599	7.8E-08
19872	Rny1	RNA, Ro-associated Y1	0.591	2.9E-04
20557	Slfn3	Schlafen Family Member 12	0.577	2.4E-05
14719	Got2	Glutamic-oxaloacetic transaminase 2	0.530	2.1E-03
12865	Cox7a1	Cytochrome c oxidase subunit VIIa polypeptide 1	0.511	6.7E-04
56338	Txnip	Thioredoxin interacting protein	0.509	6.5E-05
83554	Fstl3	Follistatin-like 3	0.498	1.3E-05
12925	Crip1	Cysteine-rich protein 1	0.496	1.0E-06
319178	Hist1h2bb	Histone cluster 1, h2bb	0.486	3.3E-05
11303	Abca1	ATP-binding cassette, sub-family A (ABC1), member 1	0.485	1.7E-06
17873	Gadd45b	Growth arrest and DNA-damage-inducible, beta	0.467	8.2E-03
19725	Rfx2	Regulatory factor x,2	0.449	9.6E-04
66929	Asf1b	ASF1 anti-silencing function 1 homolog B	0.447	2.4E-05
70893	Glb1l3	Galactosidase, beta 1 like 3	0.441	9.1E-05
11830	Aqp5	Aquaporin 5	0.439	7.1E-03
30794	Pdlim4	PDZ and LIM domain 4	0.437	1.2E-05
19784	Rprl2	Ribonuclease P RNA-like 2	0.427	8.3E-04
75316	Taf1d	TATA box binding protein (Tbp)-associated factor, RNA polymerase I, D	0.418	4.5E-05
11927	Atox1	ATX1 (antioxidant protein 1) homolog 1	0.399	3.6E-05
109900	Asl	Argininosuccinatelyase	0.397	1.3E-06
108015	Chrnb4	Cholinergic receptor, nicotinic, beta polypeptide 4	0.393	1.5E-03
15561	Htr3a	5-hydroxytryptamine (serotonin) receptor 3A	0.388	6.0E-04
319155	Hist1h4c	Histone cluster 1, H4c	0.387	1.1E-03
12306	Anxa2	Annexin A2	0.387	4.3E-04
11854	Rhod	Ras homolog gene family, member D	0.386	2.8E-04
18129	Notch2	Notch gene homolog 2 (Drosophila)	0.385	1.6E-05
77462	Tmem116	Transmembrane protein 116	0.380	9.3E-05
70807	Arrdc2	Arrestin domain containing 2	0.378	1.1E-03
11853	Rhoc	Ras homolog gene family, member C	0.372	2.9E-05
227292	Ctdsp1	CTD small phosphatase 1	0.372	1.6E-03
171209	Accn3	Amiloride-sensitive cation channel 3	0.371	2.8E-03
69875	Ndufa11	NADH dehydrogenase (ubiquinone) 1 alpha subcomplex 11	0.370	3.6E-06
65972	Ifi30	Interferon, gamma-inducible protein 30	0.368	5.5E-04
74760	Rab3il1	RAB3A interacting protein (rabin3)-like 1	0.363	1.2E-04
263406	Plekhg3	Pleckstrin homology domain containing, family G member 3	0.362	6.9E-05
71660	Rarres2	Retinoic acid receptor responder (tazarotene induced) 2	0.355	6.1E-03
212974	Athl1	ATH1, acid trehalase-like 1	0.354	1.2E-03
12036	Bcat2	Branched chain amino-acid transaminase 2	0.354	1.2E-05
68337	Crip2	Cysteine-rich protein 2	0.350	5.4E-03
22228	Ucp2	Uncoupling protein 2	0.349	3.8E-03
66230	Mrps28	Mitochondrial ribosomal protein S28	0.349	1.2E-03

**Table 2:** Genes down-regulated in retinas of diabetic mice.

ID	Symbol	Gene name	LogFc	P value
12182	Bst1	Bone marrow stromal cell antigen	−0.495	9.0E-06
219257	Pcdh20	Protocadherin	−0.440	4.0E-04
319554	Idi1	Isopentenyl-diphosphate delta isomerase 1	−0.438	6.8E-05
12293	Cacna2d1	Calcium channel, voltage-dependent, alpha 2/delta subunit 1	−0.437	4.0E-04
331487	Uprt	Uracil phosphoribosyltransferase (FUR1) homolog	−0.435	8.6E-04
83924	Gpr137b	G protein-coupled receptor 137B	−0.407	1.9E-03
66917	Chordc1	Cysteine and histidine-rich domain (CHORD) containing	−0.399	3.3E-04
66637	Tsen15	tRNA splicing endonuclease 15 homolog	−0.398	7.9E-03
20383	Srsf3	Terine/arginine-rich splicing factor 3	−0.390	5.4E-03
26557	Homer2	Homer homolog 2	−0.390	1.3E-03
67681	Mrpl18	Mitochondrial ribosomal protein L18	−0.383	9.5E-03
217951	Tmem196	Transmembrane protein	−0.383	9.9E-04
105727	Slc38a1	Solute carrier family 38, member 1	−0.362	4.7E-04
74182	Gpcpd1	Glycerophosphocholine phosphodiesterase GDE1 homolog	−0.356	5.7E-06
229279	Hnrnpa3	Heterogeneous nuclear ribonucleoprotein A3	−0.352	9.5E-05
21333	Tac1	Tachykinin, precursor 1	−0.352	5.2E-03
14405	Gabrg1	Gamma-aminobutyric acid (GABA) A receptor, gamma 1	−0.352	4.2E-03
102910	Armcx4	Armadillo repeat containing, X-linked 4	−0.351	9.7E-05
59058	Bhlhe22	Basic helix-loop-helix family, member e22	−0.351	1.9E-03
14401	Gabrb2	Gamma-aminobutyric acid (GABA) A receptor, beta 2	−0.349	5.6E-05
57329	Otor	Otoraplin	−0.345	1.8E-03
70930	Nol8	Nucleolar protein 8	−0.343	3.1E-04
70620	Ube2v2	Ubiquitin-conjugating enzyme E2 variant 2	−0.342	9.7E-04
331487	Uprt	Uracil phosphoribosyltransferase (FUR1) homolog	−0.342	1.2E-03
56353	Rybp	RING1 and YY1 binding protein	−0.341	9.6E-03
71599	Senp8	SUMO/sentrin specific peptidase family member 8	−0.339	8.9E-03
15289	Hmgb1	High mobility group box 1	−0.334	6.3E-03
15505	Hsph1	Heat shock 105 kDa/110 kDa protein	−0.333	1.1E-03
93739	Gabarapl2	Gamma-aminobutyric acid (GABA) A receptor-associated protein-like 2	−0.331	7.1E-03
668923	Zfp442	Zinc finger protein 442	−0.328	6.0E-03
72289	Malat1	Metastasis associated lung adenocarcinoma transcript 1	−0.325	1.1E-04
14009	Etv1	Ets variant 1	−0.325	6.7E-03
11798	Xiap	Apoptotic suppressor protein	−0.323	2.5E-04
17968	Ncam2	Neural cell adhesion molecule 2	−0.320	6.1E-04
76184	Abca6	ATP-binding cassette, sub-family A (ABC1), member 6	−0.314	1.4E-03
12300	Cacng2	Calcium channel, voltage-dependent, gamma subunit 2	−0.314	4.8E-03
109905	Rap1a	RAP1A, member of RAS oncogene family	−0.314	8.5E-04
71206	Katnal2	Katanin p60 subunit A-like 2	−0.312	4.3E-03
20541	Slc8a1	Solute carrier family 8, member 1	−0.312	3.1E-03
14417	Gad2	Glutamate decarboxylase 2	−0.311	6.6E-03
14823	Grm8	Glutamate receptor, metabotropic 8	−0.310	2.0E-03
14799	Gria1	Glutamate receptor, ionotropic, AMPA 1	−0.310	2.8E-04
19128	Pros1	Protein S	−0.309	1.1E-03
320772	Mdga2	MAM domain containing glycosylphosphatidylinositol anchor 2	−0.307	2.9E-04
243382	Ppm1k	Protein phosphatase 1K	−0.306	1.5E-03
246229	Bivm	Basic, immunoglobulin-like variable motif containing	−0.305	8.1E-05
228942	Cbln4	Cerebellin 4	−0.304	1.4E-03
11658	Alcam	Activated leukocyte cell adhesion molecule	−0.303	1.8E-03
18718	Pip4k2a	Phosphatidylinositol-5-phosphate 4-kinase, type II, alpha	−0.302	2.9E-04
18231	Nxph1	Neurexophilin 1	−0.302	8.0E-04

**Table 3:** Gene enrichment analysis of genes differentially expressed in retinas of diabetic mice.

Ontology	GO ID	GO Term	*P* value (P<1E-03)
**Down-regulated genes**			
MF	GO:0003723	RNA binding	6.18E-08
MF	GO:0004971	Alpha-amino-3-hydroxy-5-methyl-4 isoxazole propionate selective glutamate receptor activity	1.38E-05
MF	GO:0005488	Binding	2.8E-05
MF	GO:0022891	Substrate-specific transporter activity	2.1E-04
MF	GO:0005313	L-glutamate transporter activity	2.7E-04
MF	GO:0046943	Carboxylic acid transporter activity	4.8E-04
BP	GO:0007268	Synaptic transmission	2.4E-06
BP	GO:0007268	Transmission of nerve impulse	2.98E-06
BP	GO:0035637	Multicellular organismal signaling	2.98E-06
BP	GO:0015931	Nucleobase-containing compound transport	1.0E-04
BP	GO:0044237	Cellular metabolic process	1.0E-04
BP	GO:0050657	Nucleic acid transport	1.9E-04
CC	GO:0043005	Neuron projection	3.1E-07
CC	GO:0045202	Synapse	5.5E-06
CC	GO:0005622	Intracellular	5.6E-06
CC	GO:0044456	Synapse part	5.9E-06
CC	GO:0032279	Asymmetric synapse	2.2E-05
CC	GO:0044424	Intracellular part	2.4E-05
**Up-regulated genes**			
MF	GO:0003735	Structural constituent of ribosome	8.1E-05
MF	GO:0015078	Hydrogen ion transmembrane transporter activity	3.2E-04
MF	GO:0004364	Glutathione transferase activity	6.6E-04
MF	GO:0019843	rRNA binding	7.7E-04
BP	GO:0006979	Response to oxidative stress	6.3E-05
BP	GO:0007589	Body fluid secretion	1.4E-04
BP	GO:0016049	Cell growth	4.6E-04
BP	GO:0034599	Cellular response to oxidative stress	6.3E-04
BP	GO:0090208	Positive regulation of triglyceride metabolic process	7.2E-04
BP	GO:0070301	Cellular response to hydrogen peroxide	8.5E-04
CC	GO:0005840	Ribosome	1.4E-07
CC	GO:0070469	Respiratory chain	5.8E-06
CC	GO:0022626	Cytosolic ribosome	1.0E-05
CC	GO:0044429	Mitochondrial part	1.3E-05
CC	GO:0044444	Cytoplasmic part	1.3E-05
CC	GO:0005743	Mitochondrial inner membrane	1.4E-05

Only the six most significant gene ontology (GO) terms in each category are shown (MF, molecular function; BP, biological process; CC, cellular compartment).

**Figure 11:**
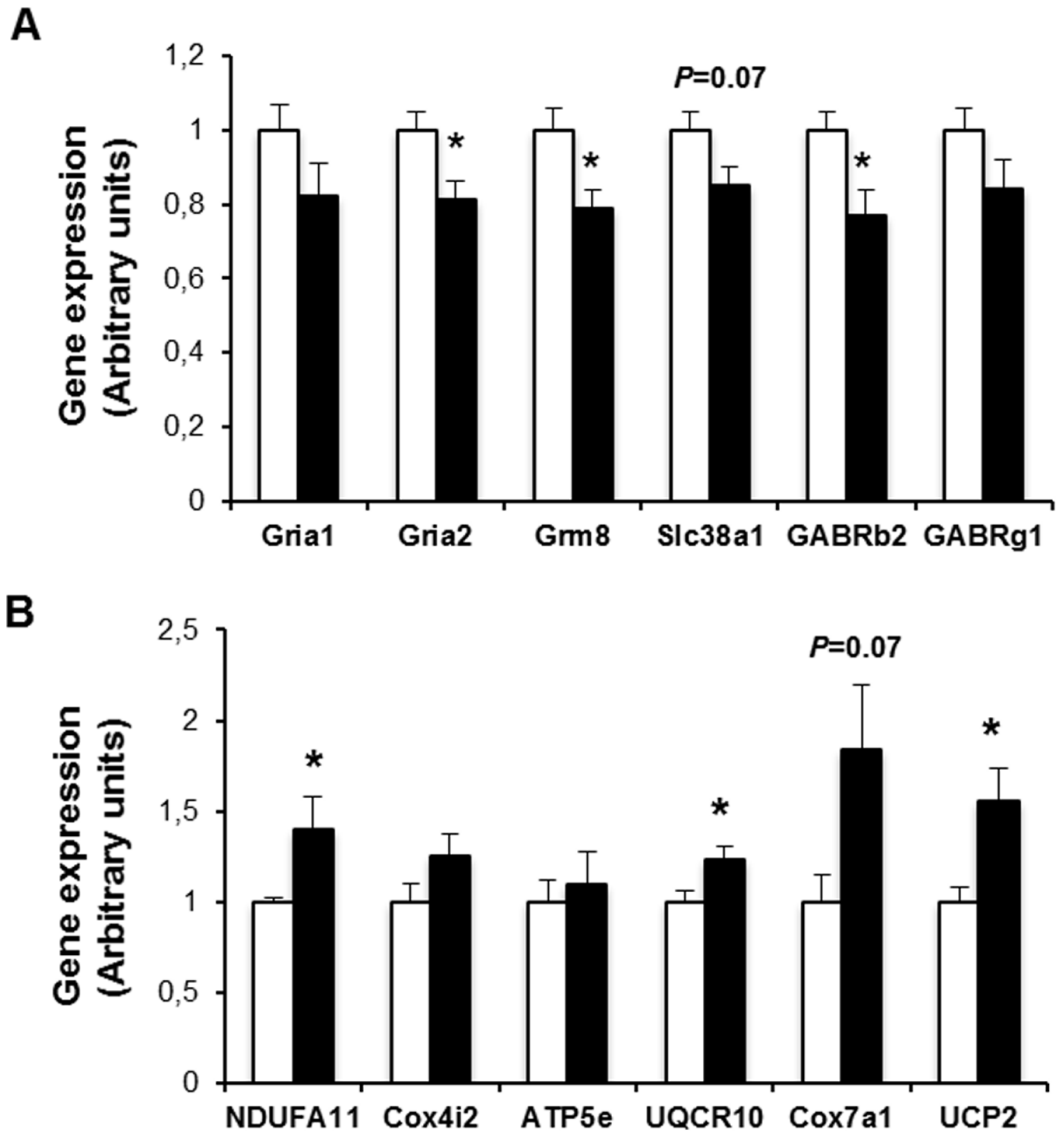
Relative expression of genes involved neurotransmission (A) and mitochondrial function (B) in retinas of diabetic mice (black bars) and control non-diabetic mice (white bars) was assessed by real-time quantitative PCR. Results are expressed as mean ± SEM, n = 9–10/group. *p<0.05.

Real time quantitative PCR confirmed an aproximately 20% reduction in the expression of genes related to neurotransmission, such as inotropic glutamate receptor-1 and -2 (Gria-1 and Gria-2), metabotropic glutamate receptor 8 (Grm8), glutamine transporter (Slc38a1) or the subunits β2 and γ1 of the gamma-aminobutyric acid A receptor (Figure 11A). These results suggest that glutamate signaling and metabolism is altered in diabetic mice.

Interestingly, we have also found that some mitochondrial genes, most of them encoding for proteins of the respiratory chain, were mildly increased in retinas of diabetic mice (Figure 11B). A parallel increase in the expression of *UCP2*, a protein involved in the control of mitochondria-derived reactive oxygen species (ROS) production, was observed (Figure 11B).

## Discussion

Neurodegeneration is an early event and plays a crucial role in the pathogenesis of DR. In fact, the main features of retinal neurodegeneration (apoptosis and glial activation) have been found in the retina of diabetic donors without any microcirculatory abnormalities appearing in the ophthalmologic examinations performed during the year before death [6], [29]. In addition, there are several pieces of evidence to suggest that neurodegeneration participates in early microvascular abnormalities that occur in DR [9]–[11], [13], [15], [30]–[32]. In this regard, blood-retinal barrier (BRB) breakdown as the result of VEGF upregulation produced by glutamate-induced excitotoxicity has been well documented [9], [11], [12], and a cross-talk between neurodegeneration and vasoregression has been reported [10], [13]. In addition, the impairment of neurovascular coupling which can be detected in diabetic patients without structural abnormalities [32], [33] seems primarily mediated by ganglion cell damage [34]. Finally, it has been shown that a delayed multifocal ERG implicit time predicts the development of early microvascular abnormalities [29], [35]–[37]. Therefore, it is reasonable to hypothesize that therapeutic strategies based on neuroprotection will be effective in preventing or arresting DR development. However, the morphological and functional characterization of neurodegeneration in a spontaneous diabetic model is a challenge that had to be met. In the present study we have evaluated the neurodegenerative process in retinas of db/db mice and we have found the same features that occur in retinas from diabetic donors. These findings permit us to propose the db/db mouse as a good model for DR and appropriate for testing neuroprotective drugs.

Mice are more resistant to the STZ effect and present a lower degree of retinal lesions compared to rats. Nevertheless, because of its great potential for genetic manipulation, the mouse offers a unique opportunity to study the molecular pathways involved in disease development. In the present study we have found that the spontaneous development of diabetes in db/db mice results in a progressive thicknness of the neuroretina in comparison with non-diabetic mice, mainly due to the apoptosis of the ganglion cell layer (GCL). In this regard, we found a 24% reduction in the number of cell bodies in the GCL at 8 weeks (after 4 weeks of hyperglycemia). This reduction increased at 16 weeks to 29%. These results are in agreement with those recently reported by Tang et al [22] showing that ganglion cell number and total retinal thickness are decreased in db/db mice compared with wild-type controls. In addition, it should be mentioned that, as occurs in db/db mice, GCL has been the layer with the highest rate of apoptosis in human retinas [6], [7], [38]. Noteworthy, thinning of the GCL has been found in diabetic patients with no or only minimal DR [6], [39]–[41]. Apart from the loss of cells in the GCL we also found a thinning of the ONL in db/db mice, which had already been found at 8 weeks. In this regard we demonstrated DNA nuclear fragmentation in photoreceptors by using transmision electron microscopy, thus clearly indicating the presence of apoptosis. This finding is consistent with the impairment of a-wave observed in the ERG studies.

Neural apoptosis is accompanied by changes in both types of glial cells (microglia and macroglia), the most representative being those occurring in Müller cells, the predominant type of macroglial cells. Retinal astrocytes normally express GFAP, while in Müller cells this expression is much lower. However, in diabetes an aberrant overexpression of GFAP is shown by Müller cells [42]. Cheung et al [19] have previously reported that GFAP and cleaved caspase 3 labeling were increased in the retina of db/db mice compared with non-diabetic controls. In the present study we have found a significant overexpression of GFAP at 8 weeks, thus running in parallel with the apoptotic changes. This is a significant feature that has not been found in other spontaneous diabetic mice such as the Ins2^Akita^ mouse model [43].

In order to explore whether neurodegeneration detected in homozygous db/db animals is of a genetic nature or related to hyperglycemia we examined the effect of lowering blood glucose levels on retinal neurodegeneration. We found a significant reduction of the most important features of neurodegeneration (apoptosis, glial activation, ERG abnormalities) by lowering blood glucose levels in db/db mice. Therefore, diabetes is the main reason accounting for the retinal neurodegeneration that occurs in db/db mice.

It is possible that glial activation is a consequence of neural death. In fact we have recently found the activation of the Fas/FasL death receptor pathway in the diabetic eye which can induce the secretion of pro-inflammatory cytokines, thus leading to neuroinflammation and glial activation [44]. On the other hand, Müller cells produce factors capable of modulating blood flow, vascular permeability, and cell survival. Therefore, these cells could play a primary role in accounting for neural death. Further studies to unravel the hierarchical role of apoptosis and glial activation in the neurodegenerative process of DR are needed.

We have also examined the functional consequences of retinal neurodegeneration by means of sequential ERG. The abnormalities here reported in db/db mice in b-wave and OPs (reduced amplitude and prolonged implicit time) were similar to those described in the early stages of DR in diabetic patients [45], [46]. The type of ERG abnormality could help to identify anatomically the location of retinal damage. In this regard, the OPs have been shown to be derived from the inner plexiform layers involving the axon terminals of the bipolar cells, the processes of the amacrine cells, and the dendrites of the ganglion cells [47]. By contrast, changes in b-wave amplitudes and implicit times are consistent with defects in the mid-retinal layer.

Nevertheless, ERG abnormalities in the diabetic retina are not only due to apoptosis of retinal neurons. In this regard, both glial activation and changes in blood glucose levels have been involved in the ERG abnormalities observed in diabetic patients without cellular loss or major structural damage [48], [49].

In the present study we found a progressive increase of glutamate accumulation in diabetic mice. Glutamate accumulation in extracellular space and the overactivation of glutamate receptors (“excitotoxicity”) plays an important role in retinal neurodegeneration. In fact, elevated levels of glutamate in the retina have been found in experimental models of diabetes [50]–[52], as well as in the vitreous fluid of diabetic patients [53]. Glutamate transporters are essential for keeping the extracellular glutamate concentration below neurotoxic levels [54]. In this regard, the glial GLAST, the main glutamate transporter expressed by Müller cells, is the most dominant glutamate transporter, accounting for at least 50% of glutamate uptake in the mammalian retina [55]. A reduction of GLAST expression in rat retinas with diabetes induced by STZ has been reported [56], [57]. However, to the best of our knowledge GLAST expression in db/db mice mouse has not previously been examined. We observed a significant reduction of GLAST in diabetic (db/db) mice in comparison with non-diabetic (db/+) mice. This finding could contribute to the glutamate accumulation that leads to neurodegeneration in db/db mice. It is worth mentioning that we did not find any differences in GLAST mRNA levels between diabetic and non-diabetic mice, thus suggesting that the differences in GLAST could be attributed to post-translational changes.

To the best of our knowledge this is the first transcriptomic analysis performed in the db/db model. We found a downregulation of several genes related to glutamate metabolism (glutamate receptors and glutamate transporters) in early stages of DR in db/db mouse. This finding could lead to the observed extracellular glutamate accumulation, thus inducing excitotoxicity. As previously commented, it has been reported that diabetes down-regulates genes implicated in glutamate transporter in animal models. However, the information on genes related to glutamate receptors is controversial and mainly focused in STZ-diabetes induced animal models [58]–[61]. Furthermore, we found a simultaneously upregulation of mitochondrial UCP2 that could be contemplated as a mechanism to mitigate oxidative stress. This finding links glutamate excitotoxicity and oxidative stress and suggests that a vicious cycle involving glutamate excitotoxicithy, oxidative stress and mitochondrial dynamics reported in a non-diabetic model of retinal neurodegeneration could also be applied to diabetic retina [62]–[65].

In conclusion, the db/db mouse reproduces the features of the neurodegenerative process that occurs in the human diabetic eye. Therefore, it seems an appropriate model for investigating the underlying mechanisms of diabetes-induced retinal neurodegeneration and for testing new neuroprotective drugs.
