# Correction: The db/db Mouse: A Useful Model for the Study of Diabetic Retinal Neurodegeneration

**DOI:** 10.1371/journal.pone.0106227

**Published:** 2014-08-19

**Authors:** The PLOS ONE Staff

The third author’s name is incorrect in the citation. The correct name is: Villena JA. The correct citation is: Bogdanov P, Corraliza L, Villena JA, Carvalho AR, Garcia-Arumí J, et al. (2014) The db/db Mouse: A Useful Model for the Study of Diabetic Retinal Neurodegeneration. PLoS ONE 9(5): e97302. doi:10.1371/journal.pone.0097302
